# Genomics of Complex Neurodevelopmental Disorders with Variable Epilepsy Phenotypes: A Clinical Review of Dup15q Syndrome

**DOI:** 10.3390/genes17020163

**Published:** 2026-01-30

**Authors:** Drew Thodeson, Trevor Lockard, Sookyong Koh

**Affiliations:** 1 MBBN Research, Omaha, NE 68106, USA; dthodeson@mbbnresearch.org; 2 Department of Pediatrics, Children’s Nebraska, Omaha, NE 68114, USA; trevor.lockard@unmc.edu; 3 College of Medicine, University of Nebraska Medical Center, Omaha, NE 68198, USA

**Keywords:** Dup15q syndrome, complex neurodevelopmental disorders, epilepsy, UBE3A, genomic medicine, autism spectrum disorder, developmental and epileptic encephalopathy, epigenetics, SUDEP

## Abstract

Background: Complex neurodevelopmental disorders frequently reflect multiple neurologic symptoms which have shared molecular and network level mechanisms. Advances in genomic medicine have redefined these conditions as overlapping manifestations of brain circuit dysfunction with significant variability. This review examines the intersection of genomics, epilepsy, and neurodevelopment in complex neurodevelopmental disorders, emphasizing Dup15q syndrome as a model for understanding phenotypic variability. Methods: Authors conducted a clinical (non-systematic) review of the literature based on their experience with three patients with Dup15q who responded dramatically to neurostimulation. We synthesized current literature on genomic mechanisms underlying complex neurodevelopmental disorders focusing on Dup15q syndrome and its subtypes—int15, idic15, and mosaic idic15. We integrated clinical, electrophysiologic, and molecular data to illustrate the spectrum of epilepsy phenotypes and their mechanistic underpinnings. Results: Dup15q syndrome demonstrates marked heterogeneity in epilepsy severity and seizure semiology, reflecting variable gene dosing effects, maternal imprinting of UBE3A, and altered GABAergic signaling. While idic15 is more strongly associated with refractory epilepsy and SUDEP, both idic15 and int15 subtypes show overlapping developmental and behavioral phenotypes. There is a well-known differential response to anti-seizure medications and emerging evidence for neurostimulation and precision medicine. Conclusion: Dup15q syndrome exemplifies the convergence of genomic, neurophysiologic, and developmental pathways in epilepsy. As genomic discovery expands, precision therapies will increasingly rely on collaborative research networks. Understanding the genomic architecture of Dup15q syndrome may inform personalized strategies for epilepsy treatment and prevention.

## 1. Introduction

Complex neurodevelopmental disorders (CNDD) represent a heterogenous group of neuropsychiatric disorders [1]. Recent advances in genomic diagnostics have yielded many monogenic causes of rare forms of CNDD. Universally, CNDD affects one or more crucial domains of human development: cognition, language, motor, and social [2]. Many children and adults with CNDD have autism spectrum disorder (ASD), intellectual disability (ID), or global developmental delay (GDD). Often all three of these conditions are present in the same individual. Frequently, learning disabilities, attention deficits, and neuropsychological conditions are also present [1,3,4]. We suggest that these disorders are best characterized by co-occurring brain network disorders that likely share similar neurological substrates and pathways. Epilepsy or susceptibility to seizure is more commonly the rule than the exception. In fact, variable epilepsy presentations and multiple seizure semiology may occur within the same person with CNDD. This review explores the neurological complexity of CNDD using chromosome 15q duplication (Dup15q) syndrome as an illustrative genomic case study. Our aims are to (1) explore the genomic mechanisms underlying CNDD and epilepsy; (2) emphasize the high prevalence of epilepsy in CNDD and illustrate the variability of seizure phenotypes; and (3) discuss Dup15q syndrome as an exemplary disorder of phenotypic pleiotropy within CNDD.

## 2. Genomic Mechanisms Underlying CNDD with Epilepsy

The paradigm of evaluation of suspected genetic CNDD has shifted from a careful description of neurological symptoms and co-occurring physical and systemic traits to genomic localization [5]. It has grown from the role of geneticists, neurodevelopmental specialists, and neurologists to involve multiple subspecialties and multi-disciplinary teams [6,7]. Accurate characterization and treatment of CNDD requires a collaborative medical home including medical subspecialists; physical, speech, and occupational therapists; social workers; school and work advocates; and cohesive families.

Genomic diagnosis alone has undergone multiple paradigm shifts in the last two decades. From mapping the human genome in 2003 [8], over 6000 rare disorders with a suspected genetic cause have been characterized and over 4000 causative genes have been identified [9], largely thanks to accessible next-generation sequencing and the pioneering scientific teams leading the research. In 2023, 900 monogenic forms of epilepsy and increased seizure susceptibility were identified [10], which has led to the elucidation of several mechanistic pathways linking genomic alterations to neuronal circuit dysfunction and epileptogenesis [11]. Mechanistic insights on brain structure have been achieved through the study of mTOR, GATOR complex, and similar pathways associated with malformation of cortical development (MCD) [12,13,14]. The term “channelopathies” has become common language among physicians, advocates, and families to describe commonalities in epilepsy and CNDD caused by ion channel abnormalities. This shift in language was made possible by scientific efforts elucidating the mechanisms of vital neuronal ion channels where pathogenic variants were found to be causal of epilepsy such as SCN1A, KCNQ2/KCNQ3, and others [15]. Through the meticulous studies of genes such as UBE3A, MECP2, ARX, and others, there has been further elucidation of various causative and protective mechanisms, such as chromatin remodeling, epigenetic changes, signal transduction, and synapse formation [16,17]. The influence of the environment on genetic expression is only beginning to be elucidated. In rat models with induced genetic absence epilepsy, it has been demonstrated that environmental enrichment could delay epilepsy onset and even alleviate epilepsy severity in adult rates. These changes were heritable to the next generation via an influence on micro-RNA expression [18].

## 3. Variable Clinical Spectrum of Epilepsy in CNDD

Epilepsy in CNDD is as variable and heterogenous as the monogenetic, complex genetic, and idiopathic causes of the CNDD themselves. Seizure semiology can be focal-onset, generalized-onset, mixed, or unknown. There may or may not be an attributable epilepsy syndrome. Infantile epileptic spasms syndrome (IESS) and Lennox–Gastaut Syndrome (LGS) are examples of developmental and epileptic encephalopathy (DEE) where the epileptic activity itself interferes with development of function during critical periods of brain development and contributes to severe cognitive and behavioral impairments above and beyond what might be expected from the underlying pathology alone [19]. When an epilepsy syndrome is present, the syndrome may be self-limited or evolve over time. Developmental regression is relatively common and may or may not be associated with seizures or epileptiform discharges. Electroencephalography (EEG) and magnetic resonance imaging (MRI) are pathognomonic for some yet are non-specific in most. EEG commonly evolves—some EEG patterns improve over time while some worsen. Even within specific monogenic causes, such as in SCN1A and STXBP1, there is variable genotype–phenotype correlation or extreme phenotypic pleiotropy [20,21]. Beyond individually identifiable genes, genome-wide association studies (GWAS) have shown more than 10 loci of significance for various types of epilepsy, such as 2p16.1 for childhood absence epilepsy or 4p12 for juvenile myoclonic epilepsy [22]. While some genomic results carry significant prognostic implications, there are always exceptions. Indeed, what is certain is uncertainty, and much remains unclear even given all the scientific and technological advances in genomics available today.

## 4. Dup15q Syndrome: An Illustrative Example of Phenotypic Pleiotropy

### 4.1. Genomic Basis

15q11-q13 chromosomal abnormalities have variable phenotypic presentations. Deletions cause Prader–Willi or Angelman syndromes inhibiting MECP2 action, while duplication produces Dup15q syndrome [23,24]. Dup15q syndrome is rare and mostly occurs de novo, with an incidence of 1:30,000 and prevalence of 1:5000 [23,25]. Dup15q syndrome consists of the more rare but less severe interstitial chromosome 15 duplication (int15), and the more severe and more common pseudoisodicentric or isodicentric chromosome 15 duplication (idic15) [26]. There are common breakpoints in certain copy repeats on proximal loci on 15q which cause instability and susceptibility to duplication [27].

Dup15q syndrome can be subdivided into three main variations—int15, idic15, and mosaic idic15. This molecular difference is subtle with idic15 described as a doubled and mirrored fragment extending from the p-arm and involving a more distal breakpoint than int15 [27]. Critical to all individuals with Dup15q is the duplication of the Prader–Willi Angelman Critical Region (PWACR), without which the clinical syndrome does not manifest. This region functionally is duplicated and classically idic15 causes tetrasomy while int15 causes trisomy. Copy number of the PWACR affects gamma-aminobutyric acid (GABA) receptor expression and thereby epilepsy phenotype and response to anti-seizure medications (ASMs); however, copy number and size of the duplication are not known to have significant influence on the neurobehavioral phenotype [24,27,28]. Curiously, an interstitial triplication, despite creating tetrasomy of the critical region, results in a phenotype resembling int15 rather than idic15 [27]. This suggests that there are mechanisms beyond the abnormal expression of GABA receptors driving epilepsy and epileptogenesis in affected individuals. Mosaic idic15 is also possible and associated epilepsy phenotypes are similar to that of individuals with idic15 [24].

While PWACR is critical to pathogenesis, there are other known loci that are overexpressed in Dup15q which have been found to contribute to neurodevelopmental abnormalities. One hypothesis of a mechanism contributing to epileptogenesis and neurodevelopmental disability in Dup15q is overexpression of UBE3A which causes neuroexcitability, leading to epilepsy, while impaired neurocognitive development leads to ASD [26,28,29]. Moreover, dysfunction of UBE3A is maternally imprinted, increasing the complexity of studying this specific hypothesis given the variability in paternal UBE3A silencing and maternal UBE3A imprinting [30]. The MECP2 gene also acts on the PWACR, and it has been shown that deficiency of MECP2 also causes deficiency of certain critical genes, including UBE3A and GABRB3. A complex pathway of genetic expression therefore links Rett, Angelman, and Dup15q syndromes [24]. Other overexpressed loci include GABRA5 and GABRG3 [24].

### 4.2. Clinical Features

Regardless of the genotype, individuals with Dup15q have significant speech disorders, hypotonia, stereotyped behaviors, abnormal socialization, aggression, impulsivity, and autistic tendencies. Dysmorphic features, if present, tend to be subtle and restricted to the face. MCDs or congenital anomalies are rare [28]. We define the primary manifestations of Dup15q as neurodevelopmental dysfunction, epilepsy, and hypotonia. Overall, communication and daily living skills are more impaired compared to socialization, motor skills, and adaptive behavior [28]. Individuals with Dup15q generally see global improvement and progression of behavior and adaptive skills over time, except for those with severe epilepsy [28].

#### 4.2.1. Developmental Features

Many similarities exist between interstitial and isodicentric behavior phenotypes, including a predilection for ASD, ID, and GDD. Autistic features show no statistical difference between interstitial and isodicentric groups, although there is higher reported rates of hyperactivity and behavioral issues in idic15 compared to int15 [23]. GDD is expected to have greater profundity and severity in idic15. There is no clear correlation with size of duplication and behavioral phenotype in idic15 [28]. Some of the developmental differences between int15 and idic15 are driven by more severe and higher rates of medically intractable epilepsy in individuals with idic15. This is discussed in more detail below. Interestingly, correcting for epilepsy makes the course of development similar between groups, but idic15 has more severe ID regardless of epileptic phenotype. A rare few individuals with int15 have normal intellectual function [23].

#### 4.2.2. Epilepsy Characteristics

Epilepsy occurs in over half of individuals with Dup15q. In total, 57–63% of individuals with idic15 and up to 16% of those with int15 develop epilepsy, typically in childhood [23,24]. While not the case for their int15 counterparts, individuals affected by idic15 have higher risk for epilepsy-associated mortality, including sudden unexpected death in epilepsy patients (SUDEP) and death due to status epilepticus. Epilepsy onset may occur after genomic diagnosis and commonly progresses to DEE with more complicated course seen in idic15 with multiple seizure types and more significant developmental fluctuation and regression [25]. Individuals with int15 have fewer seizure types and may have less severe epilepsy; however, these individuals may also develop a disabling DEE. Generalized tonic–clonic seizures are common; however, multiple seizure types have been documented [24]. IESS may precede other epilepsy types and classical hypsarrhythmia on EEG and exclusive infantile spasms may portend favorable short-term treatment response [24,31]. Regardless, more than 90% of individuals with IESS associated with Dup15q syndrome develop subsequent epilepsy [24]. Neuroimaging abnormalities have been reported; however, magnetic resonance imaging studies do not tend to show abnormalities [32]. EEG abnormalities include diffuse encephalopathic patterns, multifocal and/or generalized spike and wave discharges, and dysregulated sleep [27].

The rate of SUDEP in Dup15q syndrome is estimated at 2.6/1000 patient years, and epilepsy is an independent risk for mortality in individuals with idic15. Over 80% of cases of premature death in idic15 occur in individuals with epilepsy [27]. Overall, this frequency is similar to that of patients with epilepsy and ID or patients with treatment refractory epilepsy, highlighting the need for aggressive treatment in patients with idic15. Anecdotally, some idic15 deaths occurred after GABAergic ASM administration, which prompted the community to consider these medications higher risk due to the disruption of the GABA-A receptor in Dup15q syndrome. One study analyzed various risk factors, however, and found only non-ambulatory status to be a significant comorbidity, increasing the odds of SUDEP in Dup15q syndrome, possibly because non-ambulatory status is a proxy for significant neurodevelopmental impairment [27].

#### 4.2.3. Hypotonia

Central hypotonia is usually present from birth, with variable severity ranging from feeding difficulty as a newborn to delayed gross motor milestones [25]. Joint laxity is common later in life and may lead to injuries or chronic pain. Drooling is a frequent concern [28]. Most individuals with Dup15q syndrome learn to walk independently by 24 to 36 months of age; however, some require assistive devices for ambulation [33,34]. Feeding and adequate caloric intake is challenging in some individuals with Dup15q syndrome, requiring gastrostomy tube feeding [33]. Early assessment of motor development, careful monitoring of weight gain and feeding refusal, and signs of dysphagia should be monitored carefully and appropriate physical and occupational therapy, feeding therapy, and diagnostics employed when warranted.

### 4.3. Therapeutic Response

Dup15q syndrome treatment requires developmentally appropriate therapies for neurodevelopmental differences, careful monitoring of hypotonia and feeding issues, and appropriate ASM regimen when epilepsy is present. ASMs are not very effective and adjunctive therapy such as dietary management and neurostimulation are often used [24,35]. Valproic acid is a preferred ASM commonly used in combination with lamotrigine or levetiracetam [24,31,36]. A single case series suggests focal epilepsy associated with Dup15q syndrome may respond better than generalized epilepsy and that the presence of classic hypsarrhythmia was predictive of seizure freedom at three years follow up [31]. Another case series reported refractory epilepsy in individuals with DEE with modified or no hypsarrhythmia epilepsy [28]. Long-term outcomes are clearly variable and need further study. GABAergic ASM may worsen seizures in Dup15q syndrome. Clobazam is used with some limited efficacy [22,33].

New therapeutic approaches such as vagal nerve stimulation, responsive neurostimulation, palliative surgery, serotonin or 5-hydroxytryptamine 1A (5HT_1a_) receptor agonists, and soticlestat are currently being explored [37,38]. Soticlestat, a selective cholesterol 24-hydroxylase inhibitor, has been shown in a phase II clinical trial to decrease overall seizure frequency by 23.4% in Dup15q syndrome patients with DEE [38]. Overall outcomes in Dup15q syndrome remain highly variable, ranging from nearly normal IQ and function to GDD and death due to refractory epilepsy. We recently published a triad of cases of idic15 who had dramatic response to neurostimulation [35]. Further studies are needed to determine the efficacy of neurostimulation, as we rely on case studies and series for the majority of our evidence in individual disorders.

## 5. Broader Implications for CNDD Management

In Dup15q syndrome, like other CNDD, it is not uncommon for developmental delays to precede the onset of seizures and epilepsy. Prompt identification of neuroatypicality can lead to more prompt referral to pertinent subspecialists and vital supportive therapies. Early identification and initiation of diagnostic and therapeutic intervention is essential and improves outcomes and quality of life for individuals with Dup15q syndrome, as is true for all CNDD [33]. Commonly this identification starts with the general pediatrician and an agnostic approach to genomic evaluation of children with GDD and ID has been proposed by the American Academy of Pediatrics to improve early detection of genomic causes of CNDDs [6]. However, socio-economic factors, fear, and stigma remain a barrier to accessibility of genomic diagnostics in the 21st century even in the most developed parts of the world [39,40].

While Dup15q syndrome does not have a cure, modern genomic advances are accelerating precision medical care in monogenetic diseases [41]. In fact, whereas 15 years ago, diagnosing a monogenic condition was felt to represent the end of a diagnostic odyssey without much practical use, today, genomic testing results are actionable, researched, of intense scientific scrutiny, and have precision targets that have practical implications to people with CNDD such as MECP2, SCN1A, and others [26,42]. Neurostimulation in tuberous sclerosis is one such example, where the known multifocal epileptogenic networks are interrupted by VNS with remarkable efficacy [43]. CRISPR-Cas has been successfully used for years to treat spinal muscular atrophy, and treatment for Huntington’s Disease already shows promise in clinical trials [44]. In Dup15q syndrome, there is currently avid exploration of UBE3AE as a precision target for therapy [26].

## 6. Conclusions

Genomics, epilepsy, and neurodevelopment in CNDD have created a shared biological language in which genetic variation shapes both the vulnerability and resilience of the developing brain. Through advances in sequencing, network modeling, and molecular neurobiology, it has become clear that epilepsy and seizure susceptibility do not occur in isolation. It is a dynamic manifestation of broader neurodevelopmental circuitry dysfunction.

Dup15q syndrome serves as a powerful model for understanding this complexity. Its variable epilepsy phenotypes highlight the intricate relationship between gene dosage, imprinting, and synaptic regulation. The lessons learned extend well beyond chromosome 15, offering insight into how dosage sensitive genes and network level changes produce diverse and heterogenous manifestations across individuals with CNDD. Dup15q not only produces a genomic syndrome but also a conceptual framework for exploring genotype–phenotype variability across multiple brain network functions.

The path forward requires the integration of genomics with neurophysiology, clinical observation, and data science through collaboration. Multicenter studies and shared data repositories can accelerate discovery of precision biomarkers, inform prognostic modeling, and guide development of personalized interventions. Family advocacy groups have informed these collaborations and are becoming more visible in the rare disease space. As precision medicine advances, the goal is not simply to classify CNDD more accurately but to transform genomic knowledge into actionable, individualized therapies that improve quality of life for affected children and families.

## Data Availability

No new data were created or analyzed in this study.

## Abbreviations

The following abbreviations are used in this manuscript:

CNDD	Complex neurodevelopmental disorders
ASD/ID	Autism Spectrum Disorder/Intellectual Disability
GDD	Global Developmental Delay
IESS	Infantile Epileptic Spasm Syndrome
LGS	Lennox–Gastaut Syndrome
DEE	Developmental and Epileptic Encephalopathy
ASM	Anti-Seizure Medication
Dup15q	Chromosome 15q duplication
Idic15	Pseudoisodicentric or isodicentric chromosome 15 duplication
Int15	interstitial chromosome 15 duplication
mTOR	Mammalian target for rapamycin
GATOR complex	GTPase activating protein (GAP) activity toward recombinant activating genes (RAGs) complex
MCD	Malformation of Cortical Development
SCN1A	Sodium voltage-gated channel alpha subunit 1
MECP2	Methyl-CpG binding protein 2
KCNQ2/ KCNQ3	Voltage-gated potassium channel subunits 2 and 3
UBE3A	Ubiquitin protein ligase E3A
ARX	Aristaless-related homeobox, X-linked
STXBP1	Syntaxin-binding protein 1
PWACR	Prader–Willi Angelman Critical Region
GABRB3	Beta-3 subunit of the GABA-A receptor
GABRA5	Alpha-5 subunit of the GABA-A receptor
GABRG3	Gamma-3 subunit of the GABA-A receptor
SUDEP	Sudden Unexpected Death in Epilepsy Patients
5HT_1A_	Serotonin or 5-hydroxytryptamine 1A
